# Scarlatina

**Published:** 1853-10

**Authors:** 


*Scarlatina.*—Dr. Hester, of Reading, Penn., mentions the treatment of seventy-two cases of scarlatina, which he conducted during the last year. They were all treated by laxatives, when required, which was usually at the commencement, and the unrestrained use of hydrochloric acid, diluted with water, and sweetened, except in one case, where bronchitis demanded antimonials. Fifteen or twenty drops of the acid were put into half a pint of water, well sweetened with white sugar, and allowed *ad libitum.* It was a pleasant drink to the patient. No other general treatment was resorted to. Dr. H. attributes considerable efficiency to its use, yet not as a specific to be relied on, without other means. He thinks that, when early used, it has a salutary effect on the local inflammation of the throat. Externally, to the throat, he uses counter-irritants, preceded by leeching, in severe cases. A liniment of ol. tereb and ol. oliv., was applied every three or four hours, and the sloughy ulcers of the throat were satisfactorily treated by solution nit. arg. of two scruples to the ounce of water, applied once a day by means of a nicely trimmed sponge upon a bent whalebone handle. The nostrils and throat must be kept open by the occasional use of sage tea, acidulated with vinegar.—*Iowa Med. Jour.*

